# The Impact of Social Supports on the Excessive Alcohol Use of the Middle-Aged Adults in South Korea: Do All Types of Social Supports Have Positive Effects on Excessive Alcohol Users?

**DOI:** 10.3390/ijerph191912624

**Published:** 2022-10-03

**Authors:** Seong-Jun Maeng, Kwang-Hyun Kim, Jun-Hyeok Kang

**Affiliations:** 1 Department of Social Welfare, Sungkyunkwan University, Seoul 03063, Korea; 2 Department of Social Welfare, Seoul National University, Seoul 08826, Korea; 3 Department of Addiction Rehabilitation and Social Welfare, Eulji University, Seongnam 13135, Korea

**Keywords:** South Korean middle-aged adults, social support, excessive alcohol use

## Abstract

The purpose of the study is to discuss the necessity of interventions on excessive alcohol use among middle-aged adult Koreans and attempt to investigate the effect of social supports including family support and friend support on excessive alcohol use. To achieve these goals, a self-administered online survey was conducted on middle-aged adult Koreans from 40 to 59 years old sampled through the convenience allocation extraction method, with responses of a total of 767 samples analyzed. The results from the analysis was that the support of the family reduced excessive alcohol use, whereas the support of friends provoked excessive alcohol use. Based on these results, the necessity of a distinction in the different types of social supports for interventions in excessive alcohol use was revealed. In addition, several practical and political implications for the alleviation of excessive alcohol use among middle-aged adults are recommended.

## 1. Introduction

Many people enjoy drinking alcohol all over the world. According to the ”Global Status Report on Alcohol and Health 2018” by the World Health Organization (WHO), 43% of the world’s population (about 2.348 billion people aged 15 or older) drink alcohol in 2016, and 12.5% (about 683 million people) had drunk alcohol in the past 12 months [1] (p. 39). This means that more than half of the world’s adult population has experienced drinking.

However, many Koreans rely on alcohol beyond merely enjoying it. In 2016, the prevalence of alcohol use disorders (AUD) in Korea was 13.9%, which is more than double the world average (5.1%) [1] (pp. 72, 332). According to the National Mental Health Survey 2021 of Korea, in particular, the lifetime prevalence of alcohol use disorder was 11.6%, higher than that of other mental disorders (the prevalence of nicotine use disorder was 9.5%, that of anxiety disorder was 9.3%, and that of depression disorder was 7.7%) [2]. In addition, the proportion of people who drink “more than four times a week” increased significantly after the outbreak of the COVID-19 pandemic [3].

Excessive alcohol use can cause a range of physical and mental problems. Some studies demonstrated that people with AUD are about 2.6 times more likely to have suicidal thoughts than the normal drinking group [4]. Moreover, excessive individual alcohol use may lead to social problems such as drunk driving [5] and increased social costs [6].

Excessive alcohol use can occur in all age groups; in Korea, however, middle-aged adults have been especially vulnerable to it. According to the “Korean Health Statistics 2019”, the ‘high-risk drinking rate’ was higher in individuals in their 40s (15.3%) and 50s (13.9%) than in other age groups [7]. Considering that the middle-aged adults are vulnerable to hazardous drinking due to declined physical function [8], more studies and social discussions are needed to prevent hazardous drinking by middle-aged adults. However, there are lack of interventions to deal with excessive alcohol use in Korea. According to data from the Korean Ministry of Health and Welfare and the Health Insurance Review and Assessment Service, only about 4% of patients with alcoholism received formal treatments. In particular, the registration rate of excessive drinkers in the Addiction Management Integrated Support Center, which provides services for AUD, was only 0.68% in 2018, and even continued to decline to only 0.59% in 2020 [9]. Additionally, in the stress appraisal and coping model, people evaluate and cope with stressful situations according to the amount and quality of personal and environmental resources they possess [10]. Considering that stress is a major predictor of dangerous drinking, it is necessary to consider environmental factors, as well as personal factors, to effectively intervene in excessive alcohol use. However, the interventions in excessive alcohol use in Korea tend to focus mainly on individual factors [11].

‘Social supports’ are frequently regarded as a protective factor for drinking problems. Social supports refer to positive emotional and material resources such as love and friendship that can be obtained through social relationships [12]. Several previous studies have reported that social supports from family and friends reduced alcohol consumption and facilitated rehabilitation [13,14,15,16,17,18,19,20,21]. However, previous studies overlooked the ambivalence of these social supports. The effects of social supports can vary depending on the types of providers, so, if social supports offer an alcoholic drink, the recipients are more likely to consume it.

Thus, it is necessary to verify what kinds of social supports fit the social supports hypothesis and identify what kinds of social interventions are needed. As a result, this study developed the research questions regarding whether the support of family and friends, representative social support variables, affect the excessive alcohol use of middle-aged adults. Therefore, the research questions in this study are as follows. First, does family support for the middle-aged adults reduce excessive alcohol use? Second, for the middle-aged adults, does friend support reinforce excessive alcohol use?

## 2. Background

### 2.1. Excessive Alcohol Use

A moderate alcohol consumption may have some advantages, such as stress reduction. Excessive alcohol consumption, however, results in negative effects on physical and mental health and further causes problems in one’s daily social life [22].

In particular, alcohol use disorder (AUD) is defined as the impaired ability to stop or control one’s alcohol use, despite negative consequences in daily life due to it [23]. The DSM-IV, published by the American Psychological Association (APA), distinguished “alcohol abuse” and “alcohol dependency” as separate diagnoses, but since the publication of the DSM-5 in 2013, these were incorporated into the concept of AUD [24,25]. However, evaluating the risks of alcohol use only based on AUD criteria is limited since alcohol consumption occurs on a continuum and can develop continuously [21]. In particular, the diagnostic criteria for AUD tend to be limited to visually and clearly distinguished targets, so it is difficult to identify excessive alcohol use in primary healthcare settings [26].

To overcome this limitation, various attempts to develop screening tools to conveniently identify excessive alcohol consumption were made. For example, the MAST (Michigan Alcoholism Screening Test) and the CAGE are known as simple and effective tools [27,28,29]. In 1989, the WHO also developed the Alcohol Use Disorders Identification Test (AUDIT), which can detect excessive alcohol use easily and early on [30]. Due to the high level of sensitivity and convenience, the AUDIT has been adapted and utilized in various countries, and its practicality and validity have been demonstrated in a number of empirical studies [31,32,33,34,35]. In Korea, the AUDIT-K, a localized version of AUDIT for the Koran context, was developed by Lee et al. [26] and has shown high reliability and validity in previous studies [36,37,38].

There are various factors associated with excessive alcohol consumption. First, women are less likely to experience an alcohol problem than men since women usually perceive more social sanctions for drinking alcohol and do not have characteristics related to excessive alcohol consumption, such as aggressiveness and antisociality [39]. Meanwhile, divorced or single adults [40] and people with lower income and educational levels [41,42,43] tend to experience excessive alcohol use. Contrary to the idea that stress increases alcohol consumption, according to the ‘stress appraisal and coping model’ by Lazarus and Folkman [44], the type of stress coping strategy is a more important factor for excessive alcohol consumption than stress itself. The model hypothesized that all people experience certain kinds of stress and the perceptions of it impact more. Based on the model, Park and colleagues [45] demonstrated that the impact of stress itself was not a significant factor for alcohol consumption, whereas an active stress coping strategy had a significant impact on the problem.

### 2.2. Social Supports and Excessive Alcohol Use

A human belongs to society and the life pattern known to be determined through various social interactions [8]. Social supports are positive resources that can be obtained from social relationships, such as those with one’s family, friends, and acquaintances, especially when one faces problems [39]. A typical model related to social support and human quality of life is the ‘main effect model,’ which assumes that the amount of perceived social support gained from social relationships affects an individual’s physical and mental health, as well as their life satisfaction [46]. This model has been substantiated through many empirical studies [47,48,49], and some studies [50,51,52] have reported that social supports also affect health behaviors, such as smoking and alcohol use.

Family support, a representative type of social support, is generally known as a protective factor for excessive alcohol use. Family support means emotional and material support provided by family members to other family members, such as empathizing with concerns of family members or providing materials to family members. According to previous studies, family conflict and cohesion are associated with alcohol consumption [53]. Families of AUD patients often have negative or dysfunctional communication patterns, and their spouses express dissatisfaction [54]. One previous study [55] also demonstrated that the families of recurrent AUD patients had lower family cohesion and more conflicts than families of those who recovered. In this way, a positive family function may be not only a protective factor for excessive alcohol use but also a strong predictor for recovery [21].

According to family system theory, family problems are revealed by ‘cyclic causality’ [56] (p. 368). For example, the husband may claim to drink because of his wife’s nagging, while the wife may claim that the husband’s drinking problems cause the conflicts they experience. On the other hand, a positive interaction can occur between an individual trying to stop drinking alcohol and other family members supporting them. Thus, family supports can create a “ripple effect” on the life of an excessive drinker. In addition, previous studies [57,58] reported that family supports have a buffering effect that lowers the risk of the recurrence of AUD.

Friend support, another one of the representative types of social support, refers to the emotional and material support that an individual provides to a friend, such as paying attention to a friend, providing courage, and providing materials. Friend support is deeply related to excessive alcohol use as well. A number of empirical studies [15,16,20,59,60] demonstrated that support from friends is an important predictor of reduced alcohol use and recovery from AUD. Furthermore, friend support is reported as a factor that lowers the risk of recurrence of AUD [13].

However, if a friend supporting an individual drinks alcohol excessively, the support from this person may not reduce alcohol use. Therefore, it is more important to identify the types of friends providing social support and their drinking behaviors. A study on the friendships of patients with AUD after rehabilitation showed that the level of friendships with non-drinkers increased, while that of drinkers decreased after rehabilitation [61]. Gordon [59] also reported that support from non-drinking friends enhanced the rehabilitation of AUD patients.

In Korea, middle-aged adults tend to spend more leisure time with their friends than with their families [62]. In addition, drinking is the most popular type of leisure activity in Korea [63]. From a social learning theory perspective, the level of alcohol consumption among the middle-aged adults may be affected by this culture [64,65,66,67]. Empirical studies [68,69] reported that frequent social gatherings may increase the likelihood of excessive alcohol consumption. Additionally, from the view of social network theory, drinking culture can be easily spread through friends [70].

## 3. Materials and Methods

### 3.1. Data and Procedure

In this study, data from the “2021 Middle-Aged Adults Mental Health Survey” were used. This survey was commissioned by the ‘Institute for Life and Culture of Sogang University’ from ‘Dataspring,’ a research company, and the survey period was from 10 to 22 October 2021. The samples were collected through conviction quota sampling. Specifically, the study was introduced to middle-aged adult Koreans aged 40–59 who joined the online site (PanelNow) of Dataspring. The middle-aged adults who have agreed to participate in the study and responded to the self-administered online survey. A total of 1025 people completed this survey, and the responses from a total of 767 people were analyzed, excluding cases with missing variables. The researchers had access to use the data for research purposes and gained the approval of the Institutional Research Board. Previous studies using this data were a studies on the relationship between depression and Internet addiction among middle-aged women, conducted by Kang and Cha [71], and a study on the moderating effect of stress-coping in the relationship between family support and excessive alcohol consumption among middle-aged adults conducted by Maeng and Kang [38].

### 3.2. Measures

The level of social support (independent variables) was measured using the Perceived Social Supports Scale developed by Park [72]. The scale consists of a total of 25 questions, including “They (family or friends) all make me feel loved and cared for” and “They (family or friends) all respect and generally accept my opinion.” Each question was answered on a five-point Likert scale (from 1 = “not at all” to 5 = “very much so”). In this survey, the reliability (Cronbach’s α) of the social support from family measurement was 0.979 and that of the social support from friends measurement was 0.967.

The level of excessive alcohol use, a dependent variable, was measured using the AUDIT-K developed by Lee [26], which is widely used in clinical settings and research in Korea. AUDIT-K is a Korean version of AUDIT, and its validity and reliability have been verified in the previous study [26]. The AUDIT-K consists of a total of ten questions, with eight questions related to the frequency of and motivations for drinking, including “How often do you drink?” and “How often do you not remember the last night because of drinking?”. Each question was measured on a five-point Likert scale (from 0 = “none” to 4 = “almost daily”). Drinking-related injuries and advised experiences for excessive drinking were measured on a three-point Likert scale (0 = “none,” 2 = “experienced before but in the past year,” and 4 = “experienced in the past year”), and questions were, “Have you or your relatives ever been hurt?” and “Have any relatives, friends, or doctors been worried about you for drinking or recommended you to quit drinking?”. Although the cut-off points varied by gender, age, and country, it was generally considered that there was excessive alcohol use when the total score was 8 or higher [73]. In this study, the reliability (Cronbach’s α) of excessive alcohol use measurement was 0.892.

As for the control variables, gender, age, education level, average household income, marital status, and active stress response were selected among the factors reported to affect dangerous drinking in previous studies [11,73,74,75,76]. Gender was dummy-treated as 0 = “female” and 1 = “male,” and age was measured numerically. The education level was measured as 1 = “high school graduate or lower,” 2 = “(specialized) college graduate,” and 3 = “graduate school or higher,” and the average monthly income of households was measured as 1= “2 million won to 3 million won (approximately USD 1,470 to 2200),” 2 = “3 million won to 4 million won (approximately USD 2200 to 2930),” 3 = “4 million won to 5 million won (approximately USD 2930 to 3660),” and 4 = “5 million won or more. (approximately USD 3660 or more)” Marital status was dummyized to 0 = “unmarried” and 1 = “married.” Active stress coping was measured using ‘the way of coping checklist’ developed by Lazarus and Folkman [44] and modified and supplemented by Jung [77]. The Stress Coping Ability Scale consists of ten active stress coping questions and ten passive stress coping questions. Each question was answered using a five-point Likert scale (from 1 = “not at all” to 5 = “very much”). In this study, the ten active stress response questions were utilized and a higher score indicates more active stress. The reliability (Cronbach’s α) of the measurement was 0.870.

### 3.3. Data Analysis

In this study, the following analysis was conducted to address the research questions derived from theoretical evidence and empirical research. First, a frequency analysis and descriptive statistical analysis were conducted to examine the demographic and sociological characteristics of the samples and the characteristics of the variables. After that, to address the research questions, three models were analyzed through OLS (ordinary least squares) multiple regression analysis. Model 1 attempted to verify the effect of family supports on excessive alcohol use and Model 2 attempted to examine the effect of friend supports on excessive alcohol use. Finally, in Model 3, the effects of both family supports and friend supports on excessive alcohol use were examined.

## 4. Results

The descriptive characteristics for the variables are reported in Table 1. The sample consisted of 333 of male and 434 female participants. With regards to marital status, 565 individuals were married and 202 were single. The average age was 48.53 years old (SD = 5.69). The average educational level of participants was 1.96 (SD = 0.55). Their average monthly income was 2.84. In 2021, the standard median income in Korea was about 1.82 million won for single-person households, 3.08 million won for two-person households, 3.98 million won for three-person households, and 4.87 million won for four-person households. The average scores of active stress coping, family supports, friend supports, and excessive alcohol use were 36.00 (SD = 5.53), 94.71 (SD = 18.31), 86.69 (SD = 15.82), and 10.20 (SD = 8.00), respectively.

The researcher performed a linear regression analysis to examine the effects of the participants’ demographic backgrounds, family supports, and friend supports on their excessive alcohol use. First, the value of the VIF (variance inflection factor), examining multicollinearity, was found to be 2.259, which is lower than 2.3, indicating that there was no multicollinearity problem in any of the models.

This is presented in Table 2. First, in Model 1, sex (B = 5.17, *p* < 0.001) and family supports (B = −0.06, *p* < 0.05) were found to be significantly associated with excessive alcohol use (Adjusted R^2^ = 0.14, *p* < 0.001).

Second, in Model 2, sex (B = 5.24, *p* < 0.001), active stress coping (B = −0.33, *p* < 0.001), and friend supports (B = 0.05, *p* < 0.05) were found to be significantly associated with excessive alcohol use (Adjusted R^2^ = 0.13, *p* < 0.001).

Finally, in Model 3, sex (B = 5.29, *p* < 0.001), active stress coping (B = −0.21, *p* < 0.01), family supports (B = −0.10, *p* < 0.001), and friend supports (B = 0.10, *p* < 0.001) were found to be significantly associated with excessive alcohol use (Adjusted R^2^ = 0.16, *p* < 0.001). Since family supports and friend supports are subconcepts of social supports, theoretically, interaction between the two variables can occur, so it is necessary to be cautious of applying both variables into single model. However, multicollinearity was not discovered according to the VIF, so both variables were put into the model and analyzed. As a result of the analysis, there was no significant difference in the direction and B value of family supports and friend supports on excessive alcohol use, and the effect of the interaction between the two variables was found to be insufficient. In other words, it was found that women are less likely to engage in excessive alcohol use problems than men, and the more actively they cope with stress, the lower the level of excessive alcohol use. In particular, the level of family supports lowered the level of excessive alcohol use, while the level of friend supports increased the level of excessive alcohol use.

## 5. Discussion

This study examined the effect of social supports on excessive alcohol use among middle-aged adult Koreans. In particular, the main focus of this study was on examining the impact of two types of social supports: family supports and friend supports.

Verifying the first research question, whether family supports lower the level of excessive alcohol use for the middle-aged adults, the level of excessive alcohol use decreased with the family supports. This result is consistent with the excessive alcohol use of previous studies [14,21,53,54,57], suggesting that family supports may be a protective factor for excessive alcohol use.

The second research question, whether friend supports raised the level of excessive alcohol use for the middle-aged adults, was also verified. Unlike the results of previous studies [15,16,20,59,60], friend supports for the middle-aged adults increased the level of excessive alcohol use. This result suggests that friend supports may not always be a protective factor for excessive alcohol use. This may be the result of learning through friends as suggested by social learning theory. According to the social network theory suggesting the homophily effects, this result may be caused by the interactions between similar people. In addition, since this study is not a study using longitudinal data, more attention should be paid to making causal inference. Thus, it is necessary to consider both points of view for the interpretation of the results. However, it can be carefully speculated that in Korean culture, middle-aged adults often spend their leisure time with friends, and consuming alcohol with them may have affected the result. Therefore, it is necessary to simultaneously consider the leisure culture of Korean society when investigating the relationship between the social supports of the middle-aged adults and their excessive alcohol use.

### 5.1. Limitations

This study analyzed the effect of two types of social supports—family supports and friend supports—on excessive alcohol use by considering the unique leisure culture of the middle-aged adults in Korea. However, there are some limitations in this study. First, since the sample sizes are small and collected through convenience allocation extraction sampling, which has lower representability than probability sampling, the results should be generalized carefully. A future study on the relationship between social supports and excessive alcohol consumption may be recommended to utilize a larger sample through probability sampling. Second, since drinking status of friends and family members can affect individual alcohol use, it should be controlled in the analysis model. However, it was not controlled due to the limitations of the data in this study. In future studies, the drinking state of friends and family members should be controlled in the analysis model. Third, social supports refer to positive resources that can be obtained from various social relationships, such as family, friends, and acquaintances. However, in this study, the social supports that can be obtained from social relationships besides family and friends were not included. The middle-aged adults form social relationships through various activities, such as professional and religious activities, as well as their family and friends, so social supports from these other relationships should be considered as well in the future studies.

### 5.2. Practical Implications

When counseling and intervention are conducted to help individuals recover from excessive alcohol use, families are encouraged to actively participate in the recovery process. Considering the previous studies reviewed in this study, it seems that family is a protective factor for excessive alcohol consumption. However, in Korea, interventions in excessive alcohol use are relatively focused on individuals rather than on both individuals and families [11]. Considering the review of the previous studies, it is important for institutions in Korea to include the family as well in the intervention for excessive alcohol consumption. To enhance the participation of family, the institutions can support family gatherings linked to A.A. (Alcoholics Anonymous), which has been proven effective in the recovery from excessive alcohol use.

When intervening in excessive alcohol drinking, it is necessary to identify the friendships of excessive drinkers. Just like family supports, friend supports are valuable social resources for an individual. In particular, previous studies [16,20,60] reported that friend supports were a major factor in reducing alcohol use and predicted recovery from AUD. However, the previous studies conducted in Korea also reaported [68,69] that reported that social relationships with close people are predictors of excessive drinking from the perspective of social learning theory or social network theory. Therefore, in order to lower the excessive alcohol use of the middle-aged adults in Korea, identifying and intervening in the friendship relationships of drinkers would be a more effective approach. Specifically, a social relationship reestablishment programs are needed to redefine relationships with friends related to drinking and to further strengthen relationships with friends not related to drinking.

In terms of intervening in excessive alcohol drinking among middle-aged adults, it is necessary to consider the quality of relationships, as well as the number of family or friends they have. As discussed in this study, even though there are plenty of social relationships, excessive drinking behaviors may occur as a result of dysfunctional communication or family conflicts. Additionally, alcohol-consuming friends may provoke alcohol problems as well. Therefore, when providing an intervention for excessive alcohol use, improving communication and strengthening the cohesion of families and enhancing the relationships with non-drinking friends should be considered.

Finally, for problems derived from social relations, empowering middle-aged adult individuals as well as the interventions in social relations are necessary. For example, if an individual has strong control over drinking, even if he or she often contacts friends who enjoy drinking, he or she will not reach the level of excessive alcohol use. Therefore, activities such as self-assertive training improve the ability of individuals to express their arguments or thoughts, while interacting would be an effective intervention to prevent dangerous drinking.

## 6. Conclusions

As discussed above, middle-aged adults are more likely to engage in excessive alcohol use than any other age groups in Korea, and the level of alcohol consumption of this age group is increasing. However, studies related to excessive alcohol consumption in Korea have been conducted mainly on adolescents, the elderly, or certain occupational groups. Alcohol problems among the middle-aged adults can be extended to family problems, such as domestic violence and family dissolution. Therefore, this study demonstrated related results and suggested that more detailed future studies on the excessive alcohol consumption of the middle-aged adults.

In addition, this study examined the impacts of two distinct types of social supports, family supports and friend supports, which were demonstrated to serve as protective factors in previous studies on excessive alcohol use were examined. It was found that social supports are a stimulating factor for an individual, including the middle-aged adults. However, considering drinking and leisure culture in Korea, friend supports should be carefully considered as a positive resource for excessive alcohol use. Therefore, when intervening in excessive alcohol use with the help of social supports, it is necessary to consider various aspects of social supports.

Finally, this study demonstrated that the individual stress coping ability as well as the social supports are also an important factor for the excessive alcohol use of the middle-aged adults. Therefore, when intervening in the excessive alcohol use of middle-aged adults, individual coping capabilities should be improved simultaneously.

## Figures and Tables

**Table 1:** Descriptive statistics of the sample (n = 767).

Variables	Frequency (%)	M (SD)
Sex		
Male	333 (43.4)	
Female	434 (56.6)	
Marital Status		
Married	565 (73.7%)	
Single	202 (26.3%)	
Age		
Level of Education		48.53 (5.69)
Income		2.84 (1.20)
Active Stress Coping		36.00 (5.53)
Family Supports		94.17 (18.31)
Friend Supports		86.69 (15.82)
Excessive Alcohol Use		10.20 (8.00)

Abbreviations: M, mean; SD, standard deviation.

**Table 2:** The results of OLS regressions on excessive alcohol use (n = 767).

	Excessive Alcohol Use								
	Model 1			Model 2			Model 3		
Variables	B	SE	*t*	B	SE	*t*	B	SE	*t*
Intercept	21.86	2.92	7.48 ***	19.49	2.96	6.59 ***	20.13	2.92	6.89 ***
Covariates									
Sex (ref. Female)	5.17	0.55	9.47 ***	5.24	0.55	9.55 ***	5.29	0.54	9.78 ***
Age	−0.09	0.05	−1.92	−0.08	0.05	−1.73	−0.09	0.05	−1.80
Level of Education	−0.29	0.52	−0.57	−0.18	0.52	−0.34	−0.24	0.51	−0.47
Income	−0.27	0.26	−1.05	−0.36	0.26	−1.38	−0.32	0.26	−1.24
Marital Status (ref. Single)	0.89	0.68	1.29	0.60	0.68	0.88	1.09	0.68	1.60
Active Stress Coping	−0.09	0.07	−1.38	−0.33	0.07	−4.99 ***	−0.21	0.07	−2.89 **
Main Terms									
Family Supports	−0.06	0.02	−3.22 **				−0.10	0.02	−4.64 ***
Friend Supports				0.05	0.02	2.38 *	0.10	0.02	4.09 ***
Adjusted R^2^	0.14				0.13			0.16	
F	18.75 ***				17.97 ***			18.84 ***	

Abbreviations: B, coefficient (Bs for the OLS are interpreted as probability); SE, standard error of the coefficient; * *p* < 0.05, ** *p* < 0.01, *** *p* < 0.001.

## Data Availability

Not applicable.
